# Serodiagnostic Potential of Alpha-Enolase From *Sarcoptes scabiei* and Its Possible Role in Host-Mite Interactions

**DOI:** 10.3389/fmicb.2018.01024

**Published:** 2018-05-25

**Authors:** Jing Xu, Xing Huang, Xiaowei Dong, Yongjun Ren, Maodi Wu, Nengxing Shen, Yue Xie, Xiaobin Gu, Weiming Lai, Bo Jing, Xuerong Peng, Guangyou Yang

**Affiliations:** ^1^Department of Parasitology, College of Veterinary Medicine, Sichuan Agricultural University, Chengdu, China; ^2^Chengdu Agricultural College, Chengdu, China; ^3^Sichuan Animal Sciences Academy, Chengdu, Sichuan, China; ^4^Animal Breeding and Genetics Key Laboratory of Sichuan Province, Chengdu, China; ^5^College of Science, Sichuan Agricultural University, Ya'an, China

**Keywords:** *Sarcoptes scabiei*, enolase, embedding, immunohistochemistry, indirect ELISA, early diagnosis

## Abstract

Infestation of the epidermis with the highly contagious ectoparasite, *Sarcoptes scabiei*, causes scabies, which is characterized by intense itching, pruritus, and secondary infection. This condition affects humans, livestock, and wildlife worldwide, incurring large economic losses and reducing the quality of human life. In the present study, we cloned the alpha-enolase, a key enzyme in the glycolytic and gluconeogenesis pathways, from *S. scabiei* var. *cuniculi*, characterized it and produced soluble recombinant enolase protein (rSsc-eno). We determined the localization of Ssc-eno in isolated mites and mites in lesioned skin. The results showed that native enolase was intensely localized in the tegument of the mouthparts, the entire legs, and the whole mites' body, as well as in the gut and reproduction system. Interestingly, we found that native enolase was widely distributed in mites in lesioned skin, with obvious high protein intensity compared with isolated mites. Building on good immunoreactivity, an indirect enzyme-linked immunosorbent assay (ELISA) based on rSsc-eno showed 92% sensitivity and 95.8% specificity, compared with other indirect ELISA in this study, rSsc-eno based ELISA is better in detecting scabies in rabbits. Besides, this method can detect *S. scabiei* infection as early as 1 week post infection. Compared with other detection methods, such as traditional microscopic examination and recently published universal conventional PCR, rSsc-eno ELISA was more effective to detect early infection in rabbits. Additionally, *in vitro* incubation experiments demonstrated the concentration-dependent acaricidal activity of rabbit anti-rSsc-eno sera against larval mites, suggested its potential as a vaccine candidate.

## Introduction

Scabies caused by the permanent ectoparasite, *Sarcoptes scabiei*, is a neglected and globally prevalent contagious skin disease of humans, and many domestic and wild mammals, causing significant morbidity and mortality (Bornstein et al., 2001; Engelman et al., 2013; Hay et al., 2014). *S. scabiei* burrows into the upper layers of the skin, feeding on epidermal cells and serum (Fischer and Walton, 2014), leading to clinical signs, such as erythematous lesions, pruritus, and burrows (Arlian, 1989; Hengge et al., 2006), as well as secondary bacterial infections (Steer et al., 2009). It was estimated in 2010 that about 100 million people were infected with scabies worldwide (Hay et al., 2014); the prevalence in different regions ranges from 0.2 to 71.4% (Romani et al., 2015). Moreover, the evidence of emerging resistance to current therapeutics, such as ivermectin (Currie et al., 2004; Mounsey et al., 2009; Terada et al., 2010), permethrin and pyrethroids (Walton et al., 2000; Mounsey et al., 2008; Andriantsoanirina et al., 2014), highlighted the necessity to identify novel targets for protective intervention (new anti-parasite therapies), which requires a deeper understanding of *S. scabiei* biology and the genes with vital functions.

Enolase (2-phosphoglycerate hydratase, EC 4.2.1.11) is a ubiquitous enzyme that is involved in glycolytic and gluconeogenesis pathways (Pancholi, 2001; Rodríguez et al., 2006). In addition to its basic function as a soluble cytosolic glycolytic enzyme, enolase can function as virulence factor of streptococci (Pancholi and Fischetti, 1998; Li et al., 2013), heat shock protein of mammal cells (Sirover, 1996), or proto-oncogene regulatory protein of neuroblastoma cells (Ejeskär et al., 2005) when the protein changes its cellular localization. As a widely accepted plasminogen receptors, it is also recognized for its role in pathogenesis of trypanosomatid parasites and *Plasmodium* (Avilan et al., 2011; Ghosh and Jacobs-Lorena, 2011; Swenerton et al., 2011). In addition, enolase may also be involved in the regulation of gene transcription; cellular differentiation and growth; and the development of several organisms, such as *Giardia lamblia, Ascaris suum, Toxoplasma gondii, Naegleria fowleri*, and *Entamoeba invadens* (Segovia-Gamboa et al., 2010; Chávez-Munguía et al., 2011; Chen et al., 2011; Castillo-Romero et al., 2012; Mouveaux et al., 2014). The important roles of enolase in the life processes of various organisms suggested its potential application as candidate vaccine (Yang et al., 2010; Chen et al., 2012; Carabarin-Lima et al., 2014; Wang et al., 2014; Dutta et al., 2015) and serodiagnostic agent (Gao et al., 2016).

In *S. scabiei*, enolase was reported to exist in the *S. scabiei* var. *canis* homogenate supernatant, with relative high expression (Morgan et al., 2016), and was identified as an antigenic protein (Morgan et al., 2017). Recent evidence confirmed that enolase was the target protein of an acaricide named octadecanoic acid-3, 4-tetrahydrofuran diester and that this compound interferes with the energy metabolism of *S. scabiei* (Song et al., 2017). But beyond that, no research has been conducted on *S. scabiei* enolase, despite its potential importance in *S. scabiei*. Therefore, the aims of this study were: (i) to clone and express *S. scabiei* enolase (Ssc-eno), determine its tissue distribution in isolated mites and mites in lesioned skin; (ii) to determine the immunogenicity of recombinant Ssc-eno (rSsc-eno) and evaluate its potential as a serodiagnostic antigen for sarcoptic mange in rabbits, and to use this method to monitor the antibody level of experimentally infected rabbits; and (iii) to assess the acaricidal activity of rabbit anti-rSsc-eno sera *in vitro*.

## Materials and methods

### Ethics statement

This study was carried out in accordance with the recommendations of the animal protection law of the People's Republic of China (a draft animal protection law released on 09/18/2009). The protocol was approved by the Care and Use of Laboratory Animals of the Animal Ethics Committee of Sichuan Agricultural University (Ya'an, China) (Approval No. 2015–028).

### Parasites

The *S. scabiei* variety used in this study was derived from a clinically affected New Zealand White rabbit and then maintained in New Zealand White rabbits. The mites, a pool of adults, nymphs and larvae, were collected and stored in liquid nitrogen for RNA extraction.

### Sera

Positive rabbit sera against *S. scabiei* (50 samples) were collected from naturally infected rabbits in three rabbit farms located in Sichuan Province, China, according to two gold standards including skin lesions type and observation of the mite in skin scrapings (Casais et al., 2015). Positive rabbit sera against *Cysticercus pisiformis* (14 samples, confirmed by autopsy) and *Psoroptes ovis* var. *cuniculi* (nine samples, confirmed by visible compatible skin lesions in the ear canal and identification of *Psoroptes* mites by micrography) were also collected from farms in Sichuan Province. Negative sera (48 samples) were collected from rabbits with no presence of skin lesions from two farms without a history of mange in Sichuan Province (no presence of *C. pisiformis* was confirmed by autopsy). Half of the negative samples were used to determine the cut-off value for all iELISAs in this study, and the other half were used to test the specificity of these iELISAs.

### Expression and purification of rSsc-eno

Total RNA was extracted from pool staged sarcoptic mites and reverse transcribed into complementary DNA (cDNA) using a RevertAid™ First Strand cDNA Synthesis Kit (Thermo Scientific, USA) according to the manufacturer's instructions. Based on the annotated *S. scabiei* var. *cuniculi* transcriptome datasets (He et al., 2017a), the full-length sequence encoding mature Ssc-eno was amplified from *S. scabiei* cDNA using the primers 5′-*CGGATCC*ATGTCCATCAAAAAGATCTACGC-3′ (forward; the *Bam*HI site is in italics) and 5′-*CGAGCTC*TCAAACTGGGTGGCGGA-3′ (reverse; the *Sac*I site is in italics), and then ligated into the plasmid expression vector pET-32a(+) (Novagen, Madison, WI, USA). The recombinant protein was expressed in *Escherichia coli* and purified as described previously (Zheng et al., 2016). The eluted protein was concentrated and dialyzed with phosphate buffered saline (PBS) using Amicon Ultra Centrifugal Filter devices (Millipore, Billerica, MA, USA) according to the manufacturer's protocol. The purity of the eluted protein was detected by 12% sodium dodecyl sulfate-polyacrylamide gel electrophoresis (SDS-PAGE) and the concentration was measured using a BCA protein assay reagent (NJJCBIO, China).

### Bioinformatic analysis of the Ssc-eno coding sequence

Open Reading Frame Finder (http://www.ncbi.nlm.nih.gov/gorf/gorf.html) was used to determine the open reading frame of Ssc-eno and the deduced amino acid sequence, while SignalP 4.1 (http://www.cbs.dtu.dk/services/SignalP/) was applied to predict signal peptide. The molecular weight, isoelectric point (pI), conserved domains, and protein properties were predicted using tools on the ExPaSy web site (http://web.expasy.org/). Sequences were aligned using Clustal X software version 1.83 (Thompson et al., 1997) and the secondary structure was inferred using the YASPIN secondary structure prediction program (http://www.ibi.vu.nl/programs/yaspinwww/). Phylogenetic tree was constructed with MEGA 5·0 software (neighbor-joining method) (Tamura et al., 2011).

### Antigen and polyclonal antibody preparation

The rSsc-PYP-1, rSsc-lipocalin-2, rSsc-profilin [screened from transcriptome data of *S. scabiei* var. *cuniculi* (He et al., 2017a)] were all produced and purified similarly as rSsc-eno. All recombinant proteins were solubly expressed. The recombinant proteins were preserved at −80°C and later used to assess their serodiagnostic potential.

The polyclonal antibody against rSsc-eno was obtained as previously described (Zheng et al., 2016). Briefly, collecting rabbit serum before immunization and 2 weeks after the final injection, and subjecting to the indirect ELISA based on rSsc-eno. Finally, pre-immune serum and serum against rSsc-eno were purified using HiTrap Protein A affinity chromatography (Bio-Rad) and the IgG obtained was preserved at −80°C until use in Western blotting analyzes and immunohistochemical analyzes.

For the preparation of Anti-His sera, His-tagged proteins was expressed in plasmid expression vector pET-32a(+) (with no insert fragment) and purified, and then produced Anti-His sera using the above mentioned procedures. The obtained sera were used as a negative control in *in vitro* experiment.

### Western blotting analyzes

The total proteins of *S. scabiei* from rabbits were obtained using a mammalian protein extraction kit (CWBIO, Beijing, China). Purified rSsc-eno and total mite proteins were separated by 12% SDS-PAGE and then transferred onto nitrocellulose membranes (0.22 μm). After blocking for 2 h with 5% (w/v) skim milk (in Tris-buffered saline) at room temperature, the membranes were incubated with *S. scabiei*-infected rabbit serum and anti-rSsc-eno rabbit IgG (1:200 v/v) respectively overnight at 4°C, while non-infected rabbit serum and IgG purified from pre-immunized rabbit serum were used as negative controls. After that, the membranes were washed and incubated with horseradish peroxidase (HRP)-conjugated goat anti-rabbit IgG (1:3,000 dilutions; Boster Bio-project Co, Wuhan, China) for 1 h at 37°C. Finally, the nitrocellulose membranes were visualized using an Enhanced HRP-DAB Chromogenic Substrate Kit (Tiangen, Beijing, China).

### Preparation of paraffin sections and immunohistochemical analyzes

**Figure 1:**
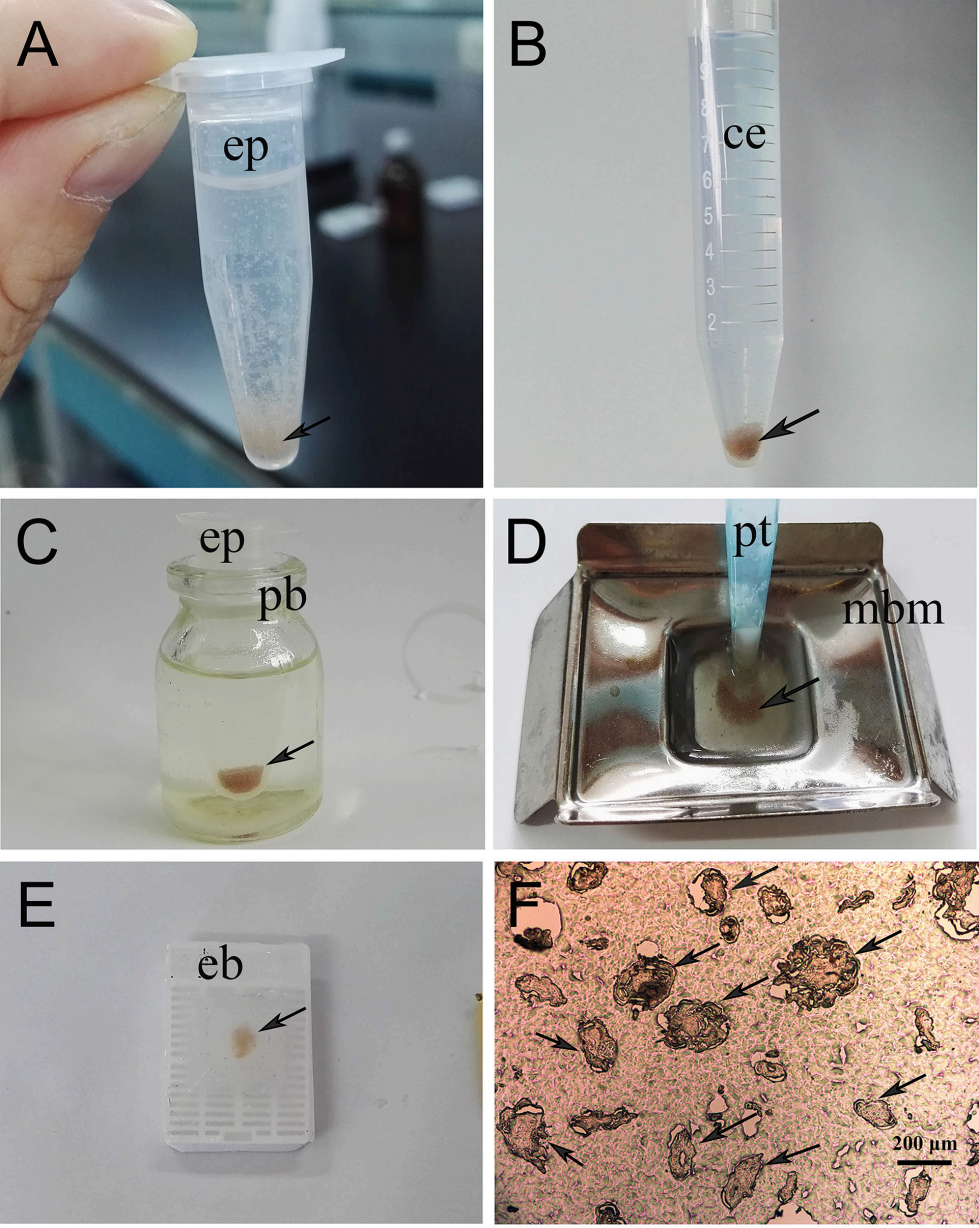
Flow chart of the direct paraffin wax embedding method for isolated scabies mites. **(A)** pooled mites fixed in 4% phosphate-buffered paraformaldehyde, the statue of mites after shaking; **(B)** dehydration and clearing, statue of pooled mites after treated with either gradient alcohol (75, 85, 95, 100, and 100%) or xylene and then standing for a few minutes; **(C)** wax immersion, the statue of pooled mites before putting into a drying oven; **(D)** preparation of paraffin wax blocks; **(E)** the resulting paraffin wax containing pooled mites after removing from its mold; **(F)** the resulting paraffin section (5-μm thickness) cut by using a rotary microtome and took photo under microscope. The arrows indicate mites. ep, Eppendorf tube; ce, centrifuge tube; pb, Penicillin Bottle; pt, peeled tip; mbm, metal base mold; eb, embedding box.

To prepare paraffin sections for immunohistochemical experiments, newly collected parasites and skin samples from *S. scabiei*-infested rabbits were used. In the present study, we developed a new method to embed isolated mites. The workflow was as follows. Firstly, the mites (20–50 mg; a pool of adults, nymphs and larvae) were fixed in 4% phosphate-buffered paraformaldehyde in a 1.5 mL Eppendorf tube for 36 h (Figure 1A). The samples were then carefully rinsed with tap water to avoid losing mites; after several washes, the samples were transferred to a 15 mL centrifuge tube and incubated in tap water overnight to clear the paraformaldehyde; the tap water was then removed as far as possible using a pipettor. Subsequently, the samples were hydrated in gradient alcohol (75, 85, 95, 100, and 100%) and made transparent using xylene (Figure 1B). The mite samples were then transferred into a 1.5 mL Eppendorf tube and 1.2 mL of melted paraffin wax was added to immerse the sample. The Eppendorf tube was placed in a Penicillin Bottle containing melted paraffin, and then placed in the drying oven at 60°C to heat the mite samples evenly (Figure 1C). It is worth noting that the mite samples sunk to the bottom of different tubes after a few minutes standing during the processes including hydrating, making transparent and soaking paraffin wax because of their relatively high density. Finally, the mite samples were transferred (using a pipettor with peeled tip preheated to 60°C) into a metal base mold containing melted paraffin wax (Figure 1D). After solidification overnight at room temperature, the paraffin wax blocks (Figure 1E) were sectioned using a rotary microtome (5 μm, Figure 1F). Skin samples were embedded using conventional methods.

For immunohistochemical analyzes, procedures were performed as described previously (Zheng et al., 2016). The stained samples were mounted with glycerol/phosphate buffer (v/v, 9:1) and a coverslip, and viewed under a fluorescence microscope (Olympus, Japan).

### Establishment of the rSsc-eno indirect ELISA

ELISAs were performed essentially as described previously (Crowther and Walker, 2009; Huang et al., 2016). Briefly, the optimal concentration of antigens and serum were determined by standard checkerboard titration procedures. The ELISA plates were coated with 100 μL of six different concentration of rSsc-eno protein (serially diluted in 0.1 M carbonate buffer, pH 9.6; ranging from 4.3 to 0.27 μg/well) overnight at 4°C. After washing, the plates were blocked with 5% skim milk for 1.5 h at 37°C and then incubated with 100 μL of twofold dilutions (ranging from 1:40 to 1:1,280) of the positive and negative sera samples for 1 h at 37°C. Subsequently, the plates were incubated with 100 μL of HRP-conjugated goat anti-rabbit IgG (Boster Bio-project Co) at 37°C for 1 h. It should be noted that the plates were washed three times with PBST (0.01 M PBS +0.05% Tween-20), this washing step occurred after each incubation, and all incubations were continuously agitated. After washing, antibody binding was determined using 100 μL of tetramethylbenzidine (Tiangen) in the dark for 15 min and the OD_450_ value was determined in a microplate reader (Thermo Scientific, Pittsburgh, PA, USA) after the reaction was stopped with 100 μL of 2 M H_2_SO_4_. The optimal working conditions were determined when the highest P/N value were given between positive and negative serum.

Under the optimized conditions, 24 negative serum samples from naïve rabbits were used to determine the cut-off value of the iELISA, which was calculated as the mean OD_450_ plus three standard deviations (SD) (Jacobson, 1998). The intra- and inter-assay repeatabilities were calculated using coefficients of variation (Coefficient of variation (CV) of raw OD) of every serum sample, essentially as previous described (Sanchez et al., 2002; Casais et al., 2015).

### Sensitivity and specificity of the rSsc-eno indirect ELISA

To further prove the feasibility of the indirect ELISA, 50 serum samples from rabbits naturally infected with *S. scabiei* were evaluated and the correct diagnostic rate was calculated based on the cut-off value. To evaluate the specificity of the indirect ELISA, cross-reactions were detected with *C. pisiformis*-positive rabbit sera and *P. ovis* var. *cuniculi*-positive rabbit sera. In addition, 24 serum samples from mange-free rabbits collected from farms without a history of mange were also tested to calculate the specificity of the indirect ELISA. Every serum sample was tested for three repeats. In summary, the sensitivity (%) of the rSsc-eno iELISA was calculated as ELISA positive × 100/true positive, and the specificity (%) was calculated as ELISA negative × 100/true negative.

### Experimental infection of rabbits with *S. scabiei* and surveillance of anti-Ssc-eno antibodies

Fifteen, 3-month-old scabies-free New Zealand White rabbits of 2.5–3 kg were purchased from the Laboratory Animal Center of Sichuan Agricultural University (Ya'an, China). Animals were kept under observation during an acclimatization period of 2 weeks and were confirmed to be clinically, parasitologically, and serologically free of sarcoptic mites. Ten rabbits (five females and five males) were infested with approximately 2,000 mixed life-cycle stage live mites on each previously shaved hind limb (foot area), dressed, and worn for 24 h (no dressings were removed mechanically by the animals). Another five rabbits were included as non-infested controls. Infestations were allowed to progress until 4 weeks post infection (PI), and the success of mite establishment was confirmed by the development of lesions in the foot area. Serum samples were collected once a week until the fourth week PI (i.e., before infection, 1 week PI, 2 weeks PI, 3 weeks PI, and 4 weeks PI). The anti-Ssc-eno antibodies of 75 serum samples (50 from the experimental group and 25 from the control group) were then detected using rSsc-eno based iELISA established in this study. Negative and positive controls were included in all plates.

### Comparison of the rSsc-eno based iELISA with other methods

For a better understanding of the utility of the rSsc-eno based iELISA, we compared it with other methods, including iELISAs based on other antigens and other published non-serodiagnostic methods.

Firstly, the optimized conditions for iELISA based on rSsc-PYP-1, rSsc-lipocalin-2, and rSsc-profilin were determined by standard checkerboard titration procedures as described for rSsc-eno based ELISA. Under the optimized condition, we determined the cut-off value for these ELISAs; then the sensitivity and specificity of these iELISAs were also determined in parallel to rSsc-eno based ELISA using the same serum samples (Table 1).

**Table 1:** Comparative evaluation of the serological assays for the diagnosis of sarcoptic mange in rabbits.

**ELISAs**	**Optimal concentration of antigen**	**Optimal serum dilution of antigens**	**Cut-off values**	**Sensitivity**	**Specificity**
Enolase^*^	1.07 μg/well	1:640	0.3961	92% (46/50)	95.8% (45/47)
PYP-1^&^	1.45 μg/well	1:640	0.2532	92% (46/50)	93.6% (44/47)
Lipocalin-2^ʬ^	1.95μg/well	1:320	0.4738	90% (45/50)	89.4% (42/47)
Profilin^ʬ^	3.33μg/well	1:640	0.3593	78% (39/50)	83.0% (39/47)

* *In this study*.

& *Our previous published data, Xu et al. (2017)*.

ʬ *Our unpublished data*.

Additionally, parallel experiments were also performed on two groups of rabbits, including 20 rabbits with clinical signs and 10 experimentally infected rabbits (infected as described above). For both groups, skin scrapings and serum samples were collected simultaneously. In particular, for the experimentally infected group, these samples were collected before infection, 1 week PI, and 2 weeks PI, respectively. Skin scrapings were used for microscopic examination and the total DNA was extracted for a universal conventional PCR (Angelone-Alasaad et al., 2015); serum samples were subjected to the rSsc-PYP-1 based iELISA and rSsc-eno based iELISA established in this study.

### *In vitro* assessment of the acaricidal activity of rabbit anti-rSsc-eno sera against larval *S. scabiei*

**Figure 2:**
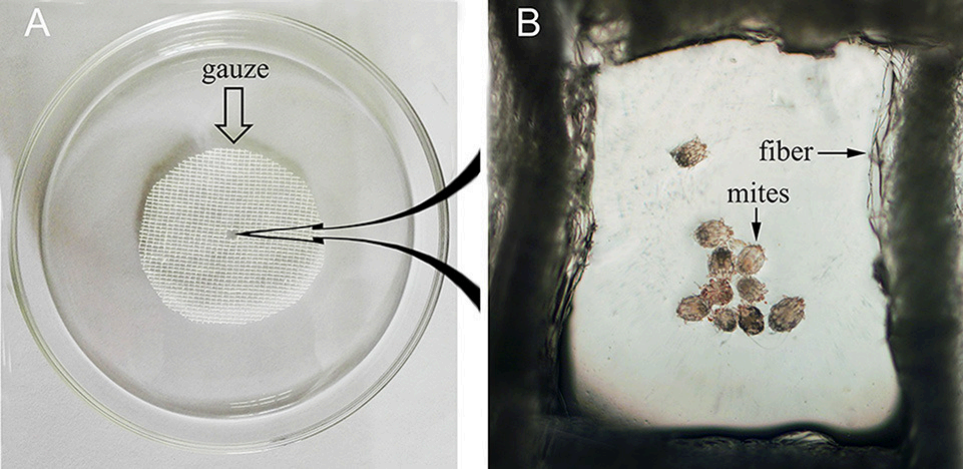
Schematic diagram of the *in vitro* incubation experiment for larval scabies mites. **(A)** Round shaped gauze. **(B)** Larval mites under the microscope (40×).

To evaluate the efficacy of anti-rSsc-eno sera against larval *S. scabiei*, larval mites (harvested within 3 h; 10 mites for each repeat, *n* = 30) were incubated in decreasing concentrations of sera diluted in PBS (1:10, 1:20, and 1:40), among which, anti-rSsc-eno sera was applied as the experimental group, and anti-His sera and negative sera were applied as negative controls; PBS served as the blank control. Each experiment involved three biological replications. The method for *in vitro* experiment was newly developed and the detailed procedures were as follows: Step 1, round gauzes (of the same size) were prepared (with the central two fibers removed) and placed in the center of a clean Petri dish (diameter, 9.0 cm; depth, 1.5 cm) (Figure 2). Step 2, for each Petri dish, ten larvae were collected in the center of the gauze using a silver needle and then sprayed uniformly until they were completely covered by the test solutions; meanwhile, the gauze was soaked thoroughly using a pipettor to add tested solutions from outside the range of the gauze. Step 3, all plates were placed in a humidity chamber (relative humidity, 75%; temperature, 25°C) and the mites were examined under a stereomicroscope every 12 h for 72 h; the test solutions were added to the gauze to keep it wet after every observation. Death of the larvae was manifested by their immobility or the lack of reactions when gently stimulated with a needle (Macchioni et al., 2004; Seddiek et al., 2013). The incubation conditions and durations of the *in vitro* experiments are summarized in Table 2. Survival rates (live mites/total mites × 100%) for each experiment were calculated at every 12 h until 72 h after incubation, data represented the results derived from three replicates.

**Table 2:** *In vitro* mortality percentage of *S. scabiei* var. *cuniculi* after treatment with solutions with different concentrations of antisera.

		**Time post treatment (h)**																	
		**12**			**24**			**36**			**48**			**60**			**72**		
		**AV ± SE**	**D/T**	**MO%**	**AV ± SE**	**D/T**	**MO%**	**AV ± SE**	**D/T**	**MO%**	**AV ± SE**	**D/T**	**MO%**	**AV ± SE**	**D/T**	**MO%**	**AV ± SE**	**D/T**	**MO%**
A	Anti-rSsc-eno sera	1.00 ± 0.58a	3/30	10	2.67 ± 0.33a	8/30	26.7	3.67 ± 0.33a	11/30	36.7	5.0 ± 0.58a	15/30	50.0	6.33 ± 0.33a	19/30	63.3	7.67 ± 0.33a	23/30	76.7
	Anti-His sera	0.67 ± 0.33a	2/30	6.7	1.67 ± 0.33ab	5/30	16.7	2.33 ± 0.33b	7/30	23.3	2.67 ± 0.33b	8/30	26.7	3.33 ± 0.33b	10/30	33.3	3.67 ± 0.33b	11/30	36.7
	Negative serum	0.33 ± 0.33a	1/30	3.3	1.00 ± 0.00b	3/30	10	1.33 ± 0.33b	4/30	13.3	2.33 ± 0.33b	7/30	23.3	2.67 ± 0.33b	8/30	26.7	3.33 ± 0.33b	10/30	33.3
B	Anti-rSsc-eno sera	0.67 ± 0.33a	2/30	6.7	1.33 ± 0.33a	4/30	13.3	2.00 ± 0.00a	6/30	20	2.67 ± 0.33a	8/30	26.7	3.33 ± 0.33a	10/30	33.3	5.67 ± 0.66a	17/30	56.7
	Anti-His sera	0.67 ± 0.33a	2/30	6.7	1.33 ± 0.33a	4/30	13.3	1.67 ± 0.33a	5/30	16.7	2.33 ± 0.33a	7/30	23.3	3.00 ± 0.00a	9/30	30	3.67 ± 0.33ab	11/30	36.7
	Negative serum	0.33 ± 0.33a	1/30	3.3	1.00 ± 0.00a	3/30	10	1.67 ± 0.33a	5/30	16.7	2.33 ± 0.33a	7/30	23.3	2.33 ± 0.33a	7/30	23.3	3.33 ± 0.33b	10/30	33.3
C	Anti-rSsc-eno sera	0.33 ± 0.33a	1/30	3.3	1.00 ± 0.33a	3/30	10	1.67 ± 0.33a	5/30	16.7	2.67 ± 0.00a	8/30	26.7	3.33 ± 0.33a	10/30	33.3	4.00 ± 0.33a	12/30	40
	Anti-His sera	0.67 ± 0.33a	2/30	6.7	1.33 ± 0.00a	4/30	13.3	1.67 ± 0.33a	5/30	16.7	2.00 ± 0.33a	6/30	20	2.67 ± 0.33a	8/30	26.7	3.33 ± 0.00a	10/30	33.3
	Negative serum	0.33 ± 0.33a	1/30	3.3	1.00 ± 0.00a	3/30	10	1.67 ± 0.33a	5/30	16.7	2.33 ± 0.33a	7/30	23.3	3.00 ± 0.00a	9/30	30	3.33 ± 0.33a	10/30	33.3
D	PBS	1.67 ± 0.19	5/30	16.7	4.67 ± 0.19	14/30	46.7	6.33 ± 0.19	19/30	63.3	6.67 ± 0.19	20/30	66.7	8.33 ± 0.19	25/30	83.3	8.67 ± 0.19	26/30	86.7

*All sera were diluted at 1:10 (A), 1:20 (B), and 1:40 (C), D represented a blank control group. Values within a column for the same group followed by different lowercase letters were significantly different (p < 0.05), while values within a column for the same group followed by the same lowercase letters were not significantly different (P > 0.05). D/T, (number of dead mites/total number of mites); MO%, Mortality%; AV ± SE, Average number of dead mites ± Standard Error*.

### Statistical analysis

For the indirect ELISA, all data were presented as the mean ± standard deviation (SD). Statistical analyzes were performed using the Mann–Whitney *U*-test for comparison between groups in the SPSS Statistics 20 package (SPSS Inc., Chicago, IL, USA). A *P*-value < 0.05 was considered statistically significant. For the *in vitro* acaricidal assay, statistical analyzes were performed by one-way ANOVA using SPSS version 20.0 (SPSS Inc., USA); Kaplan–Meier survival curves were generated with GraphPad Prism software version 5.01.

## Results

### Molecular characterization of Ssc-eno

**Figure 3:**
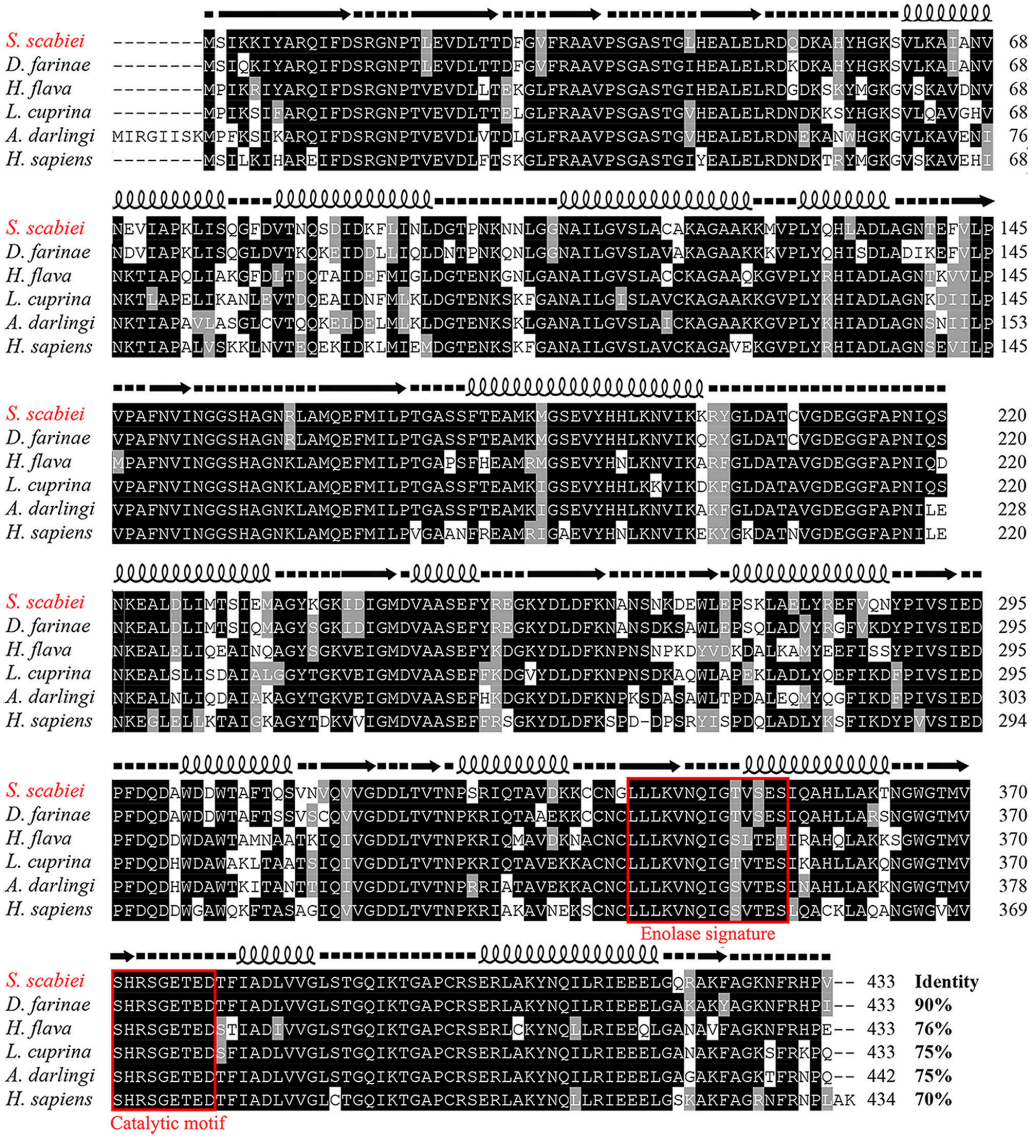
Sequence alignment of *S. scabiei* enolase (Ssc-eno). Alignments of the deduced amino acid sequence of Ssc-eno (KPM02829.1) with homologous proteins from arthropods (*Dermatophagoides farinae*: AHV90299.1; *Lucilia cuprina*: KNC30153.1; *Haemaphysalis flava*, AIS82610.1; *Anopheles darlingi*, ETN65833.1) and humans (alpha-enolase from *Homo sapiens*: AAY43128.1) were performed using Clustal X software version 1.83 and then shaded by BOXshade version 3.21. Predicted secondary structure elements of Ssc-eno, including coils, strands, and helices, are shown above the alignment as dashed lines, straight lines, and loops, respectively. The enolase signature motif and catalytic motif are enclosed in red boxes.

The full length *S. scabiei* enolase amplified in this study contained a 1,302-bp open reading frame, encoding a mature peptide of 433 amino acid residues. The obtained sequence was exactly the same as the reported enolase from *S. scabiei* var. *canis* at both the nucleotide (QR98_0012520) and amino acid level (KPM02829.1) (Rider et al., 2015). The deduced protein had a predicted molecular weight of 47.3 kDa and pI of 5.75. No signal peptide or transmembrane regions were found. A highly conserved catalytic motif 371-[SHRSGETED]-379 and enolase signature motif 341-[LLLKVNQIGTVSES]-354 were identified by online tools, confirmed the identification of Ssc-eno (Figure 3). A multiple amino acid sequence alignment revealed that Ssc-eno had the highest identity with alpha-enolase from *Dermatophagoides farinae* (90%) and *Euroglyphus maynei* (90%, partial 404 aa), followed by 74–76% overall identity with enolases from other arthropoda, 67–73% identity with those from helminths and mammalians, 62.47% identity with an enolase from *Saccharomyces cerevisiae*, and less than 60% identity with enolases from protozoa.

**Figure 4:**
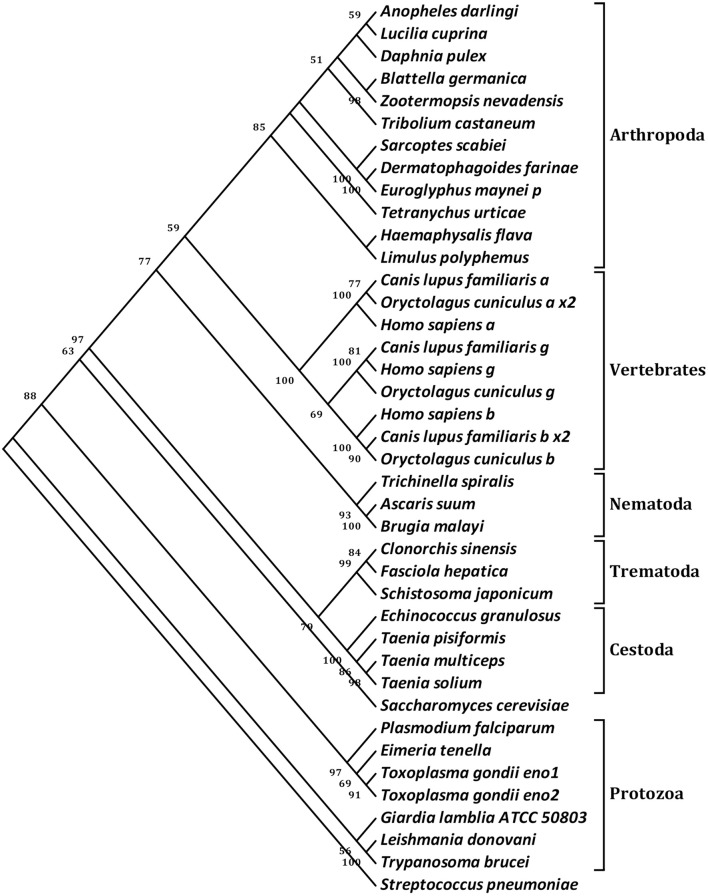
Phylogenetic relationships of *S. scabiei* enolase (Ssc-eno) with homologous enolases. The tree was constructed from a multiple sequence alignment performed using Clustal W2 and plotted using MEGA 5.10. Numbers indicate bootstrap values (>50%). The protein sequences used in the tree, with their GenBank accession numbers, are listed as follows: *Anopheles darling*, ETN65833.1; *Lucilia cuprina*, KNC30153.1; *Daphnia pulex*, EFX83276.1; *Blattella germanica*, ABC96322.1; *Zootermopsis nevadensis*, KDR20985.1; *Tribolium castaneum*, XP_975266.1; *Sarcoptes scabiei*, KPM02829.1; *Dermatophagoides farinae*, AHV90299.1; *Euroglyphus maynei p*, OTF73661.1; *Tetranychus urticae*, XP_015782335.1; *Haemaphysalis flava*, AIS82610.1; *Limulus polyphemus*, XP_013772811.1; *Canis lupus familiaris a*, XP_013972892.1; *Oryctolagus cuniculus a x2*, XP_002716189.2; *Homo sapiens a*, NP_001419.1; *Canis lupus familiaris g*, XP_003639985.1; *Homo sapiens g*, NP_001966.1; *Oryctolagus cuniculus g*, XP_002712960.1; *Homo sapiens b*, NP_001967.3; *Canis lupus familiaris b x2*, XP_005619976.1;*Oryctolagus cuniculus b*, NP_001075554.1; *Trichinella spiralis*, AAK50056.1; *Ascaris suum*, ERG79934.1; *Brugia malayi*, XP_001896281.1; *Clonorchis sinensis*, GAA51601.1; *Fasciola hepatica*, CAK47550.1; *Schistosoma japonicum*, ACV41761.1; *Echinococcus granulosus*, ACY30465.1; *Taenia pisiformis*, AGU16441.1; *Taenia multiceps*, AFJ44747.1; *Taenia solium*, AQQ11626.1; *Saccharomyces cerevisiae*, AAA88713.1; *Plasmodium falciparum*, AAA18634.1; *Eimeria tenella*, AAK38886.1; *Toxoplasma gondii eno1*, AAP24058.1; *Toxoplasma gondii eno2*, AAP24057.1; *Giardia lamblia ATCC 50803*, XP_001709336.1; *Leishmania donovani*, ACE74540.1; *Trypanosoma brucei*, AAF73201.1; *Streptococcus pneumoniae*, OBX44201.1. Notes: a (α-enolase), b (β-enolase), g (γ-enolase), x2 (isoform 2).

To probe the evolutionary position of Ssc-eno, the amino acid sequences of 40 enolases from numerous species representing most taxonomic groups were aligned and subjected to phylogenetic analysis (NJ tree) (Figure 4). In general, the results were in good agreement with traditional taxonomy, with Ssc-eno and other arthropoda species clustered into a group within the phylum Arthropoda, then with other eukaryotic species, and finally prokaryotic species, such as protozoa and bacteria. In particular, enolases from vertebrates were characterized by greater intraspecific, rather than interspecies, similarities, demonstrated as separate monophyletic branches for three different enolase isoforms (α, β, and γ) reflecting their different functions; this results was consistent with previous study (Piast et al., 2005).

### Expression and identification of rSsc-eno

**Figure 5:**
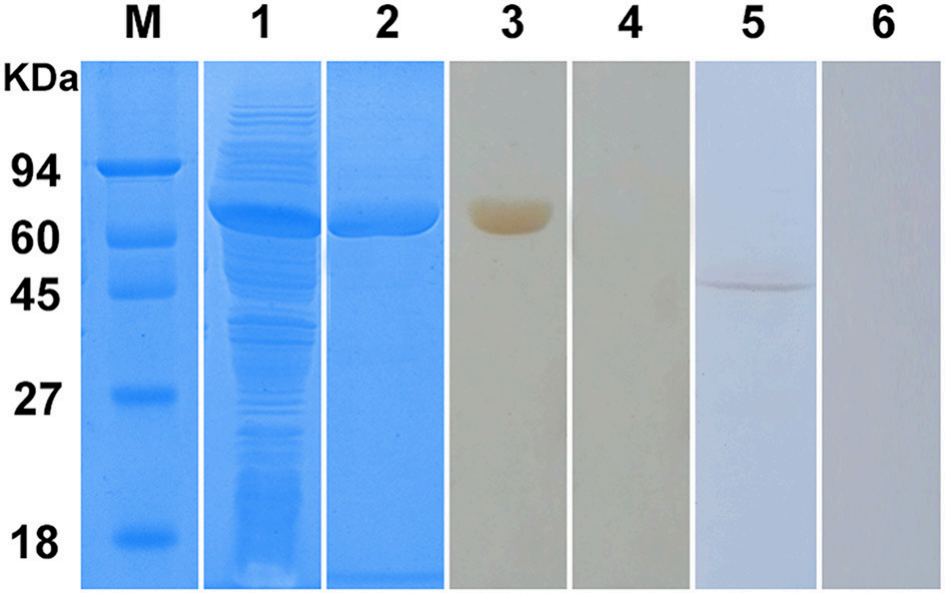
Sodium dodecyl sulfate-polyacrylamide gel electrophoresis (SDS-PAGE) and western blotting analysis of *S. scabiei* enolase (Ssc-eno). M, molecular weight markers; Lane 1, recombinant (r)Ssc-eno expressed in *E. coli* BL21 (DE3) after induction; Lane 2, purified rSsc-eno (6μg); Lane 3, purified rSsc-eno probed with serum from a rabbit naturally infected with *S. scabiei* (6μg); Lane 4, purified rSsc-eno probed with naïve rabbit serum (6μg); Lane 5, total protein from *S. scabiei* probed with IgG purified from the anti-rSsc-eno rabbit serum (30μg); Lane 6, total protein from *S. scabiei* probed with purified IgG from pre-immune rabbit serum (30μg).

The Ssc-eno gene was successfully ligated to the pET32a (+) expression vector and expressed as a soluble protein in *Escherichia coli* BL21 (DE3) cells (Figure 5, lane 1). The purified protein had an expected size of approximately 65 kDa, including an approximately 20 kDa epitope tag fusion peptide (Figure 5, lane 2). Western blotting analysis showed that a single band (approximately 65 kDa) was recognized by rabbit anti-*S. scabiei* serum (Figure 5, lane 3), which suggested strong reactivity and antigenicity of rSsc-eno. Moreover, anti-rSsc-eno IgG specifically recognized an approximately 47 kDa protein from total protein extracts of *S. scabiei* (Figure 5, lane 5), which corresponded to the size of predicted native Ssc-eno and suggested good specificity and antigenicity. No signal was recognized by serum from naïve rabbits or IgG purified from pre-immune serum (Figure 5, lane 4 and lane 6).

### Immunolocalization of native Ssc-eno

**Figure 6:**
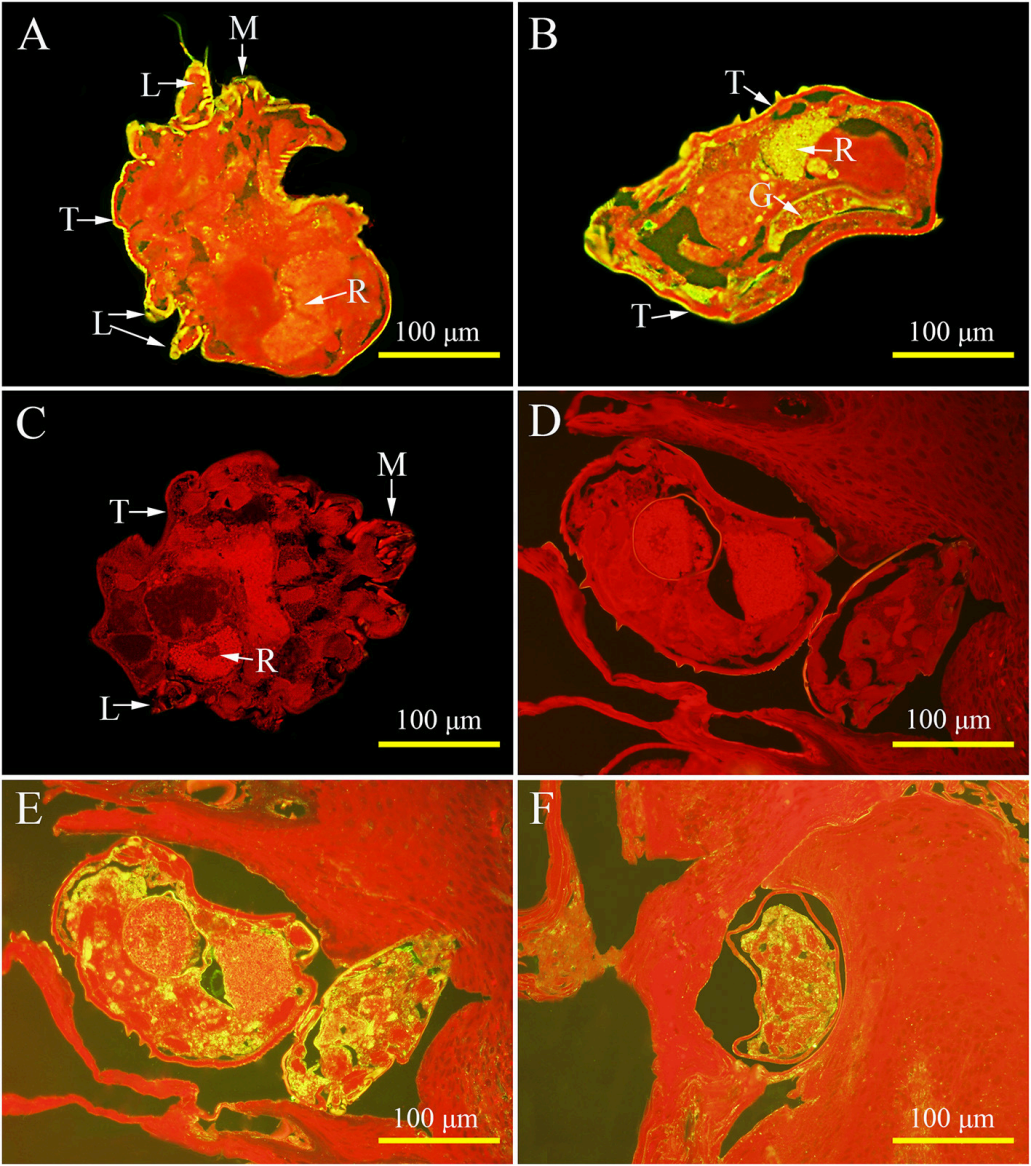
Immunolocalization of native *S. scabiei* enolase (Ssc-eno). Mites or skin samples were fixed in paraformaldehyde and embedded in paraffin. The green fluorescent color shows the location of the native Ssc-eno protein. The sections (5μm) were incubated with either rabbit anti-rSsc-eno IgG at 1:100 **(A**,**B**,**E**,**F)** or preimmune IgG at 1:100 **(C**,**D)**, diluted in phosphate buffered saline (PBS). Arrows indicate: a positive signal in the tegument around the mouthparts **(A)**; the entire legs **(A)**; the whole mites' body **(A,B)**; the gut **(B)**; and the reproduction organ **(A,B)**. No staining was observed in mites and host tissues using IgG purified from preimmune serum **(C,D)**, confirming that the detected immunolabeling was specific. T, tegument; M, mouthparts; L, legs; G, gut; R, reproduction organ.

For isolated mites, strong signals were observed on the tegument of the mouthparts, the entire legs, and the whole mites' body, as well as in the gut and reproduction system (Figures 6A,B). Interestingly, the native Ssc-eno protein was widely distributed in mites (Figure 6E) and egg during development (Figure 6F) in lesioned skin, with markedly high protein intensity compared with isolated mites. No staining was observed in mites or host tissues using IgG from pre-immune sera (Figures 6C,D).

### Establishment of the rSsc-eno based indirect enzyme-linked immunosorbent assay (iELISA)

According to the checkerboard titration protocol, the optimal concentration of antigens was 1.07 μg/well and the optimum serum dilution was 1:640 (Table 1). The cut-off value of the indirect ELISA was calculated as 0.396 (mean + 3SD): the mean absorbance was 0.242 and the standard deviation was 0.051. Therefore, serum samples with OD_450_ ≥ 0.396 were considered as positive, while OD_450_ < 0.396 were considered negative.

The coefficients of variation (CVs) of the intra-assay variation ranged from 0.55 to 4.69% (mean value 1.78%) and those of the inter-assay variation ranged from 0.86 to 7.73% (mean value 3.97%). All CVs were < 10%, indicating that the rSsc-eno indirect ELISA was repeatable and reliable.

### Sensitivity and specificity of the rSsc-eno based iELISA

**Figure 7:**
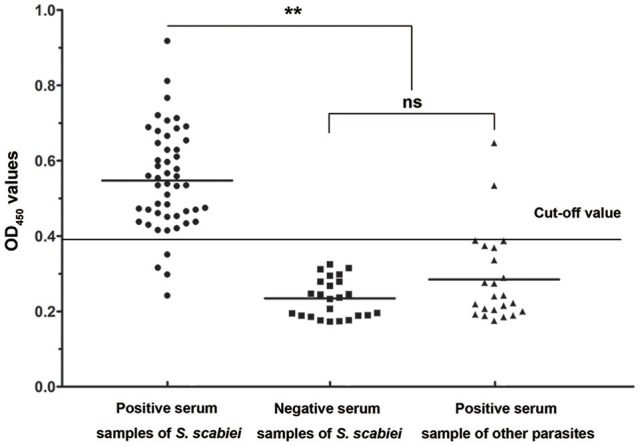
Sensitivity and specificity of the enzyme-linked immunosorbent assay (ELISA) for recombinant *S. scabiei* enolase (rSsc-eno). The horizontal line represents the cut-off value (0.396). Statistically significant differences were observed between *S. scabiei*-positive sera and the other positive sera, including *C. pisiformis*-positive sera (*n* = 14) and *P. ovis* var. *cuniculi*-positive sera (*n* = 9) (Mann-Whitney U, *z* = −6.750, *p* < 0.001), as well as between *S. scabiei*-positive sera and healthy rabbit sera (Mann–Whitney U, *z* = −5.843, *p* < 0.001). No difference was noted between healthy rabbit serum samples and the other positive serum samples (Mann–Whitney U, *z* = −1.266, *p* = 0.205).

Forty-six out of 50 sera naturally infected with sarcoptic mites tested as positive (OD_450_ > 0.396) and the sensitivity of iELISA was determined as 92% (46/50) (Figure 7, left panel). Two *P. ovis* var. *cuniculi*-positive serum samples cross-reacted with Ssc-eno, thus the specificity of the rSsc-eno ELISA was calculated as 95.8% (45/47) (Figure 7, middle and right panel). Statistical significance was observed between *S. scabiei*-positive sera and other positive sera (including *C. pisiformis*-positive rabbit sera and *P. ovis* var. *cuniculi* positive rabbit sera) (Mann–Whitney *U, z* = −6.750, *p* < 0.001), as well as between *S. scabiei*-positive sera and healthy rabbit sera (Mann–Whitney *U, z* = −5.843, *p* < 0.001). No difference was noted between healthy rabbit serum samples and the other positive serum samples (Mann–Whitney *U, z* = −1.266, *p* = 0.205).

### Surveillance of the anti-Ssc-eno antibody in rabbits experimentally infected with *S. scabiei*

**Figure 8:**
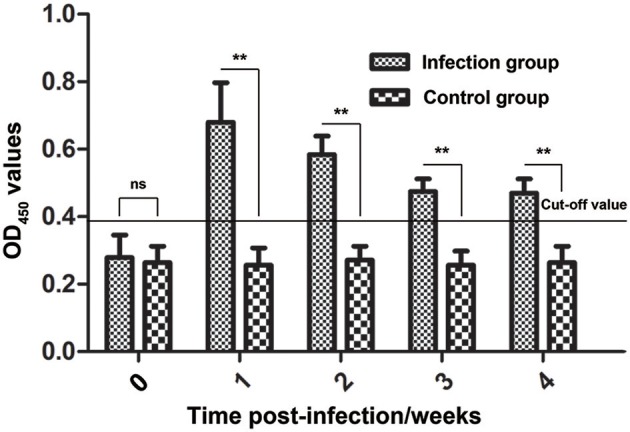
Serum antibody profiles of rabbits induced by *S. scabiei* var. *cuniculi* infection. The horizontal line indicates the cut-off value (0.396). Specific serum IgG antibodies were measured by an enzyme-linked immunosorbent assay (ELISA) for recombinant *S. scabiei* enolase (rSsc-eno) in the course of the experimental infections. Asterisks indicate statistically significant differences of anti-Ssc-eno antibody level between the infection group and the control group (^**^P < 0.01), and the error bars represent the standard deviation. “ns” indicates not significant.

During infestation, all 10 rabbits (experimental group) developed visible clinical signs at 3–7 days, with crusts starting at the site of inoculation. Figure 8 shows the changes in the anti-Ssc-eno antibody levels when rabbits were experimentally infected with *S. scabiei*. From 1 week PI until the end of the experiment, the experimental group was positive for serum antibodies against Ssc-eno (OD_450_ > 0.396). Intriguingly, the mean Ssc-eno antibody level dramatically increased at 1 week PI and then decreased in the following weeks, but still remained detectable (OD_450_ > 0.396). Significant differences in the anti-Ssc-eno antibody were observed between the experimental group and the control group from 1 week PI to 4 weeks PI (Mann–Whitney *U, P* < 0.001), while no significance was observed before infection (Mann–Whitney *U, z* = −0.367, *p* = 0.768).

### The results of rSsc-eno based iELISA compared with other detection methods

The optimal concentration of antigens and serum dilution, as well as the cut-off values of other iELISAs (rSsc-PYP-1, rSsc-lipocalin-2, and rSsc-profilin iELISA) were determined (Table 1). The sensitivities of these iELISAs were 92% (46/50), 90% (45/50), and 78% (39/50) respectively, while their specificities were 93.6% (44/47), 89.4% (42/47), and 83.0% (39/47). Due to the lower sensitivity and specificity of both rSsc-lipocalin-2 and rSsc-profilin iELISA, these methods were not applied to compare with other non-serodiagnostic methods.

In addition to comparing the rSsc-eno based iELISA with other antigen-based serodiagnostic methods, we also compared it with traditional microscopic examination and a universal conventional PCR method. This is a parallel experiment with our previously study (rSsc-PYP-1 based iELISA) and the results of the other methods have been published elsewhere (Xu et al., 2017). Briefly, for 20 clinical rabbit mange cases, only one serum could not be detected using our rSsc-eno based iELISA, whereas PCR and microscopic examination could detect all of them; for 10 experimentally infected rabbits, our iELISA was positive for all of them at 1 and 2 weeks PI, while PCR was positive for 7 rabbits (at both time points) and microscopic examination was positive for two and one rabbits at 1 and 2 weeks PI, respectively.

### The *in vitro* acaricidal activity of anti-rSsc-eno sera

**Figure 9:**
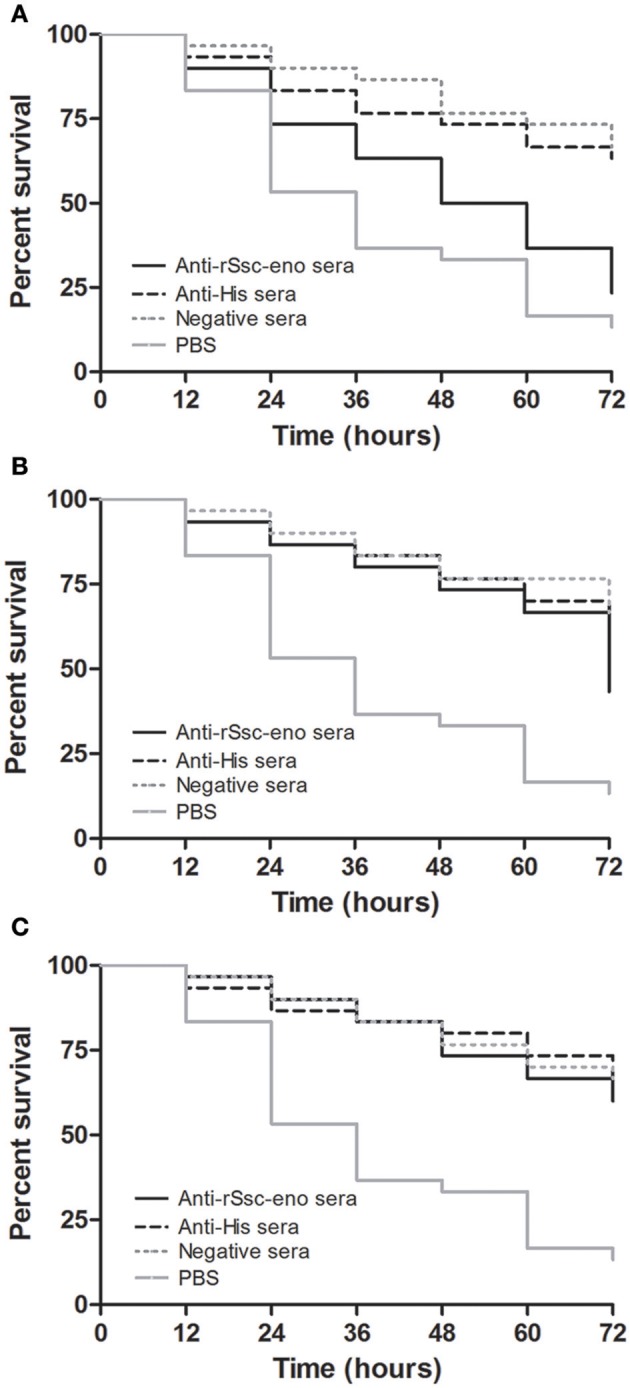
Survival curves of *S. scabiei* var. *cuniculi* incubated with rabbit anti-recombinant *S. scabiei* enolase (rSsc-eno) sera. Anti-Ssc-eno sera, anti-His sera and negative sera were diluted (in phosphate buffered saline (PBS)) into 1:10 **(A)**, 1:20 **(B)**, and 1:40 **(C)**, while PBS was used as a blank control for each concentration of sera. For each concentration (*n* = 10), three repeats were included. The *in vitro* assay was observed every 12 h for 72 h.

At the beginning of the experiment, the mites moved frequently to the inner margin of the round gauze and tried to escape from the fiber in the center of the gauze; however, the mites could not successfully stride over the barrier of gauze and stayed near the gauze fiber, with most of their body immersed in the solutions. With the *in vitro* assay, we were able to demonstrate the acaricidal activity of anti-rSsc-eno sera against scabies mites. In general, the results showed that anti-rSsc-eno serum resulted in relatively higher mortality rate compared with the negative controls over a 72 h period, and this efficacy was dose dependent (Table 2). To be specific, the highest concentration (1:10) exhibited significant higher mortality compared with negative control sera (anti-His-taged fusion protein (anti-His) sera and negative sera) since 36 h, but this efficacy was not effective to kill all mites and 7 mites were still alive to the end of the experiment; the medium concentration showed relatively more deaths (17 vs. 11 or 10) compared with negative control sera at 72 h, but the difference was only observed between anti-rSsc-eno sera and negative sera. No significance was observed at the lowest concentration (1:40) during the entire experiment. The survival curves for the different concentration of tested sera are presented in Figure 9.

## Discussion

In the present study, we cloned, expressed, and characterized Ssc-eno from *S. scabiei* var. *cuniculi*, based on a transcriptome dataset (He et al., 2017a). Phylogenetic analyzes showed that the evolutionary relationship of enolase from various species was in good agreement with traditional taxonomy, except that the isoforms of vertebrate enolases formed separate monophyletic branches because of their function. Additionally, protozoan enolases in the phylogenetic tree would benefit from the further study of their molecular evolution. Western blotting analysis showed strong reactivity and antigenicity for rSsc-eno and native Ssc-eno, suggesting that *S. scabiei* enolase is an antigenic protein with good immunogenicity. Based on these properties, the serodiagnostic potential of rSsc-eno was assessed by indirect ELISA, and then the utility of this method was determined by comparison with other sero- and non-sero- diagnostic method.

So far, there has been no report on location of native enolase in *S. scabiei*. It is interesting that the native enolase secreted onto the tegument of *S. scabiei*, because as far as we know, enolase is a key enzyme involving in glycolytic and gluconeogenesis pathways and should be located in cytoplasm. In this study, the deduced Ssc-eno protein showed no predicted peptide sequence or transmembrane domain; therefore, how the glycolytic enzyme Ssc-eno comes to be located on the tegument of *S. scabiei* remains to be determined; likewise, this is also a challenging problem for other species (Pancholi, 2001; Jong et al., 2003; Bernal et al., 2004; Avilan et al., 2011). Surface localized enolase has been characterized to be plasminogen binding protein on pathogens such as *Streptococcus mutans, Leishmania Mexicana, Fasciola hepatica* and so on (Bernal et al., 2004; Ge et al., 2004; Vanegas et al., 2007). Due to its plasminogen-binding activity, enolase promotes the tissue invasion of many pathogens within their hosts (Jong et al., 2003; Agarwal et al., 2008; Candela et al., 2009; Wang et al., 2011). Similarly, surface expressed *S. scabiei* enolase might benefit the invasion of *S. scabiei*, too. Further evidence have proposed the role of enolase in dissolving clots during feeding and preventing clotting in the tick midgut (Díaz-Martín et al., 2013), this may also explain the distribution of native Ssc-eno in gut. It is worth noting that a strong signal was observed in reproductive system, but we do not have sufficient data to explain this. For mites in lesioned skin, native enolase was widely distributed, with markedly high protein intensity compared with isolated mites, indicating its important role in host-mite interaction. But these are just speculation; further experiments are required to confirm it.

The production of paraffin sections is the main method for microstructure observation and protein localization of tissue samples. To provide better paraffin sections for the localization of native *S. scabiei* proteins, we developed a direct paraffin wax embedding method for isolated scabies mites. This method has proven to be effective in preparing paraffin sections, which could be used in different subsequent stainings, including immunostaining or hematoxylin and eosin staining. In the last few decades, studies concerning the internal structure of isolated *S. scabiei* have been relatively limited. For small tissues like mites, some researchers fixed these isolated mites using one medium before processing for paraffin embedding; agar has been the classical medium used for this purpose (double embedding method) (Zhang et al., 2012, 2013; Zheng et al., 2016; He et al., 2017b). Briefly, the isolated mites are pre-embedded in molten agar and after the agar solidified, the agar-encased tissue is subjected to routine procedures to prepare paraffin sections. However, this method has several shortcomings. For instance, some regions of the tissues do not lie flat when mounted on the slide, and then either torn away or poorly stained during histological procedures (Jones and Calabresi, 2007). More specifically, wax will infiltrate between agar and tissue because the tissue tend to shrinking during processing (Supplementary Figure 1); once sections are laid on a 42°C water bath, as the tissue rehydrate and expand more extensively than the agar and the encasing wax, resulted in the above mentioned shortcoming. Additionally, small holes and spaces around mites formed by air pockets are bad for retaining mites and subsequent proper sectioning. To obtain one section with the complete morphology of mites, many sections are needed, which is time-consuming and costly, and obtaining complete mites at different positions on one section would be even more difficult. Due to the above mentioned shortcomings, for those studies involving histological analysis, skin samples infested with *S. scabiei* were frequently used (Beckham et al., 2009; Bergstrom et al., 2009; Walton et al., 2010; Mika et al., 2012; Casais et al., 2016) instead of isolated mites. In this study, we developed a direct paraffin wax embedding method after trial and error, and effectively resolved the above-mentioned problems of pre-embedding by agar; the internal structures of the mites in the obtained sections were complete and clear, which would be useful for the subsequent staining. Over all, this method took advantage of the relatively higher density of isolated mites compared with the other reagents (different concentrations of ethyl alcohol, xylene and paraffin) used to prepare the paraffin wax block. The mites sink to the bottom under gravity without any centrifugation, thus the procedure is simple and the embedding was effective. Although the principle of this method is easy to understand, no such study has ever been conducted or reported. The developed method is both convenient and practical, and could be widely used in studies of other small tissues, such as *Psoroptes* mites (already applied in *P. ovis* var. *cuniculi*, unpublished observation) and dust mites, greatly facilitating the study of these tiny species. For example, RNA interference is now available for dust mites and scabies mites (Marr et al., 2014, 2015; Fernando et al., 2017); therefore, our new embedding method could promote the study of mite biology and gene function, revealing potential drug targets or vaccine candidates.

Clinical symptoms may be used in initial diagnosis of Sarcoptes infestation, but for definitive diagnosis, the presence of mites, their eggs, and/or fecal pellets in skin scraps is a necessary. However, very few mites are involved and no or minimal symptoms can be observed during the early infections, making it difficult to find and scrape mange lesions in humans and other mammals (Arlian and Morgan, 2017). Therefore, serodiagnostic methods would be a better choice. As the scabies mites cannot be mass cultured *in vitro*, recombinant proteins that could be recognized by circulating antibodies in an active scabies infestation would be more practical than native proteins. In this study, rSsc-eno iELISA showed a relatively high sensitivity of 92% and specificity of 95.8% compared with those iELISAs reviewed by Arlian and Morgan (2017). Besides, when compared with other antigens (rSsc-PYP-1, rSsc-lipocalin-2 and rSsc-profilin) based iELISAs in parallel, rSsc-eno iELISA has the highest specificity (95.8 vs. 93.6%, 89.4 and 83%) and sensitivity (92 vs. 92%, 90 and 78%), indicating that rSsc-eno iELISA was more appropriate to detect scabies in rabbits. Indeed, we have to admit that rSsc-eno iELISA in this study and rSsc-PYP-1 iELISA we recently developed (Xu et al., 2017) were not as good as traditional microscopic examination and newly published universal conventional PCR (Angelone-Alasaad et al., 2015) in detecting clinically suspected cases. However, for those cases in early stages of infection, it is difficult to find or scrape mange lesions to perform non-sero- diagnostics; our results showed that rSsc-eno and rSsc-PYP-1 iELISA can detect all rabbits that were experimentally infected with scabies mites as early as 1 week PI, while non-sero- diagnostic methods had lower positive rates. In combination with sensitivity and specificity, rSsc-eno iELISA was more suitable for the early diagnosis of scabies in rabbits.

According to the specific antibody level of the experimentally infected rabbits, the anti-Ssc-eno antibody significantly increased at 1-week post infection, decreased slightly in the following weeks, but still remained detectable during the experiment. This may be related to the biology of *S. scabiei* and host-mite interaction. As reviewed by Arlian and Morgan (2017), when scabies mites infect a host, they placed on the skin and secreted a clear fluid (presumably saliva) which is presumed to dissolve (lyse) the stratum corneum; as a result, the mite sinks into a depression in the skin, with the aid of legs I and II, a tunnel burrow in the stratum corneum is formed. During this progress, a humoral response was induced on the host, which resulted in circulating antibodies (which may or may not be protective). During our experimental infection, large amounts of mites initiated penetration of the skin, and many proteins and enzymes (including enolase) are excreted to the outside of the body to facilitate its penetration (Arlian and Morgan, 2017), which might have induced the dramatic increase in the corresponding antibody level. In addition, a previous study showed that the amount of trichomonads enolase increased after contact with vaginal epithelial cells (VECs) (Mundodi et al., 2008), which is consistent with our results. After the mites penetrate into the skin, the mites may no longer need so many proteins to facilitate penetration or some of these surface localized enolases (the observation of immunolocalization) are functional in other aspects such as degrade entrapped anti-Ssc-eno antibodies (Cortes et al., 2017), thus the host response to these exotic proteins may not be that strong and the anti-Ssc-eno antibody level decreased slightly.

Sheep and rabbits can develop immunity to re-infection (Rodriguez-Cadenas et al., 2010; Casais et al., 2014) with sarcoptic mites. However, these experiments were performed on hosts and the resistance may involve mixed specificities of immunoglobulins. By RNAi-mediated silencing, the biological importance of enolase was confirmed in *Clonorchis sinensis* (Wang et al., 2014), and both adult worms and metacercariae were specifically inhibited by anti-enolase rat serum (Wang et al., 2011). To ascertain whether anti-Ssc-eno antibodies could affect the growth of *S. scabiei*, the acaricidal activity of the anti-rSsc-eno antibodies was evaluated *in vitro*. We chose larval mites rather than mites in other stages, because mites in this stage will experience further development and may be more sensitive to protective immunoglobulins. The results showed that the anti-rSsc-eno sera had acaricidal activity compared with negative control groups and this efficacy was dose dependent. According to antibody trapping mechanism proposed by Cortes et al. (2017), it was hypothesized that the anti-rSsc-eno antibodies were degraded by enolase on the tegument of larvae and thus the experimental group with a higher concentration of anti-rSsc-eno antibodies showed higher mortality rates. The surface localization of *S. scabiei* enolase and acaricidal activity of anti-rSsc-eno suggested that *S. scabiei* enolase plays an important role in the *S. scabiei* invasion of host skin or in the growth of *S. scabiei*, and could be a potential vaccine candidate.

The assessment of the acaricidal activity of specific compounds against mites is usually performed *in vitro*. Filter paper (mites were placed on the filter paper, and the tested solutions were sprayed to mites) (Nong et al., 2012; Seddiek et al., 2013; Gu et al., 2014) and liquid paraffin (mites were placed into the solution diluted in liquid paraffin) (Deng et al., 2012; Hu et al., 2015; Song et al., 2017) are the two most frequently used methods. For the filter paper method, it is hard to identify and calculate the mortality under the microscope because mites move very quickly and are scattered around if the acaricidal activity of specific compounds is not very strong and mites cannot be killed quickly; additionally, the poor optical transparency of filter paper also increases the difficulty of observation. Liquid paraffin is easy to observe under the microscope; however, its viscosity diminishes the range of mites' motion. Moreover, liquid paraffin cannot be digested or absorbed in the gut, and can affect the absorption of water and the digestion of tested drugs, which may harm the mites and affect the results of the tests. In this study, we developed a round gauze-based method (see our newly developed workflow in section Materials and Methods) to assess the efficacy of chronic acaricidal contents. This method is easy to operate and observe, and can limit the motion range of sarcoptic mites without harming them, making it very practicable for *in vitro* assessment assay.

## Conclusion

In conclusion, this study characterized *S. scabiei* enolase and developed a serodiagnostic method that might contribute to the surveillance of the prevalence of sarcoptic mange in rabbits, especially diagnoses of this disease in the early stages. Furthermore, we developed a direct paraffin wax embedding method for the preparation of sections that could be used in subsequent staining; this method is simple and practical for the study of sarcoptic mites and can be promoted to other tiny mite pathogens, such as psoroptic mites, and even dust mites. The newly developed *in vitro* incubation workflow could also be applied in future *in vitro* assessment studies of *S. scabiei*. By applying these two methods in our study, we determined the localization of native Ssc-eno in *S. scabiei* and the acaricidal efficacy of polyclonal serum against it, the results revealed the possible role of Ssc-eno in host-mite interaction.

## Author contributions

JX, XH, and XD participated in the design of study and performed the data analysis; JX and XH wrote the manuscript; YR, MW, NS, YX, and XG contributed to animal care and experiments; WL, BJ, and XP participated in discussion; GY conceived of the study, participated in its design and coordination. All authors read and approved the final manuscript.

### Conflict of interest statement

The authors declare that the research was conducted in the absence of any commercial or financial relationships that could be construed as a potential conflict of interest.

## Supplementary material

Supplementary Figure 1 Sections of mites using the double embedding method. Isolated mites were collected and fixed in 1% molten agarose before routine embedding procedures. The paraffin wax was then cut into 5-μm sections by using a rotary microtome and took photo under microscope.
