# Supplementary figures and images for: Serodiagnostic Potential of Alpha-Enolase From Sarcoptes scabiei and Its Possible Role in Host-Mite Interactions
Source: Front Microbiol. 2018 May 25;9:1024. doi: 10.3389/fmicb.2018.01024 (PMC5981165; doi:10.3389/fmicb.2018.01024)

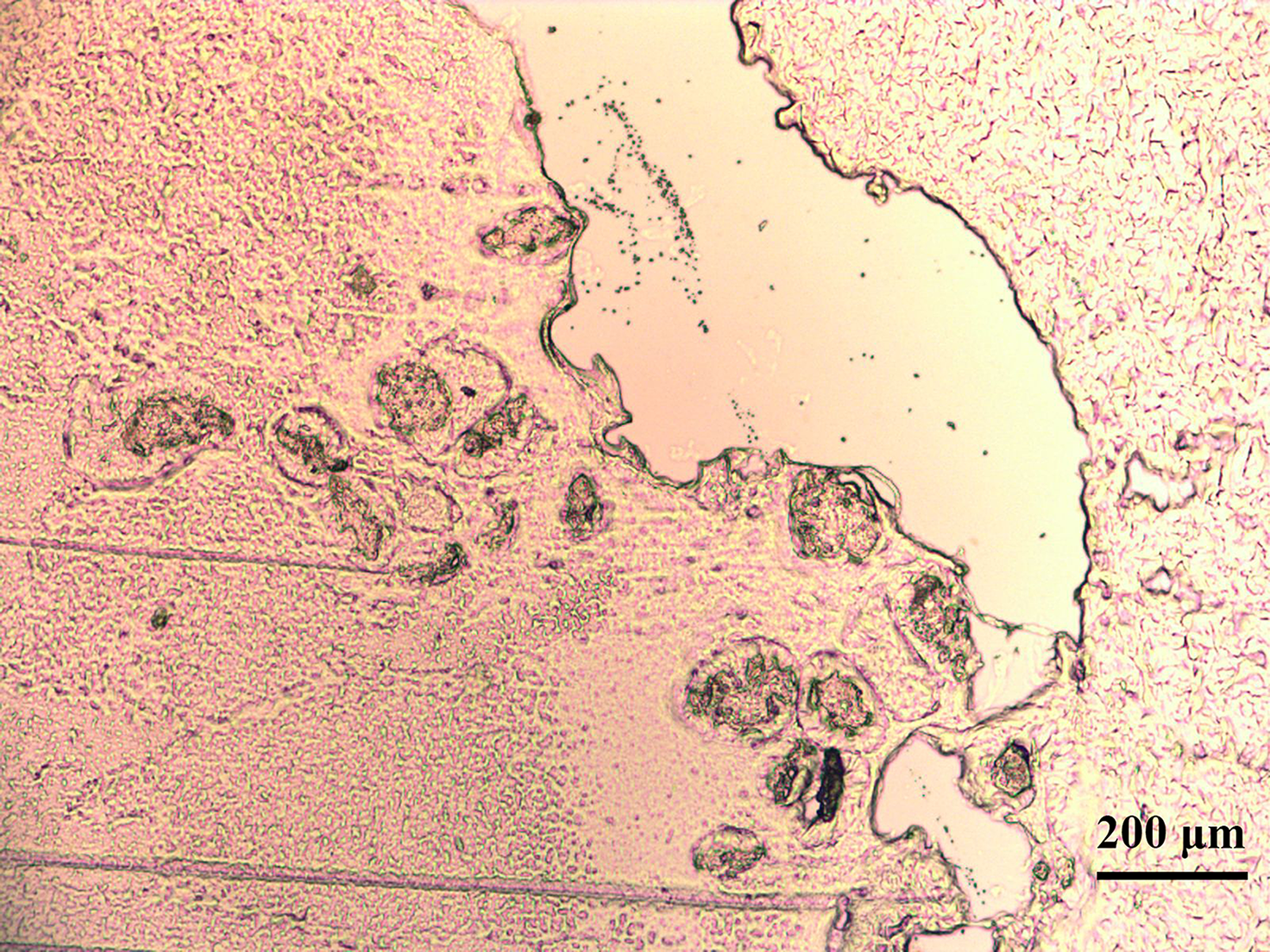

Supplement: Supplementary Figure 1 — Sections of mites using the double embedding method. Isolated mites were collected and fixed in 1% molten agarose before routine embedding procedures. The paraffin wax was then cut into 5-μm sections by using a rotary microtome and took photo under microscope. [file Image_1.TIF]
